# The forgotten needs of mothers during neonatal transfers: A quest for greater sensitivity

**DOI:** 10.4102/safp.v62i1.5091

**Published:** 2020-07-21

**Authors:** Pradeep Ashokcoomar, Raisuyah Bhagwan

**Affiliations:** 1 KwaZulu-Natal Department of Emergency Health Services, Durban, South Africa; 2 Community Health Studies, Faculty of Health Sciences, Durban University of Technology, Durban, South Africa

**Keywords:** neonates, pre-hospital, mothers, emergency medical care, clinical and emotional needs of mothers

## Abstract

**Background:**

The transfer of critically ill neonates has escalated in developing countries. This calls for greater awareness of the needs of mothers who are often overlooked while clinical attention is prioritised in relation to the neonate. The objective of the study was to understand the emotional and clinical needs of mothers during the transfer process.

**Methods:**

Using a qualitative research approach, the study sought the views of mothers who were involved in emergency transfers. In-depth interviews were held with seven mothers. In addition, data from interviews with seven neonatologists and data from focus group discussions with 35 advanced life paramedics, were included.

**Results:**

What emerged was that both the clinical and emotional needs of mothers were overlooked during the transfer which resulted in acute distress. Moreover, the study found that paramedics lacked preparedness to deal with the psychological needs of mothers and often overlooked their physiological condition as well.

**Conclusion:**

It is crucial that greater sensitivity towards both the clinical and emotional needs of mothers be prioritised during neonatal transfers.

## Introduction

Pre-hospital Emergency Medical Services (EMS) rely on dedicated professionals who must make decisions and use complex technologies in challenging field environments and under conditions of uncertainty and considerable time pressure. A recent review of the EMS literature emphasised the need for research to improve our understanding of the magnitude and threats to patient safety.^1^ The majority of existing studies examine clinical adverse events, and there is relatively little qualitative research on the contributors to safety in pre-hospital emergency care.^1,2^ The current study contributes to this gap in knowledge by building upon and further refining our understanding of the landscape of factors leading to safety events in the pre-hospital emergency care of critically ill neonates and their mothers who accompany them. Just as in hospital medicine, factors within the systems, team, child/family, and individual provider level system contribute to errors in pre-hospital emergency care.^3^

The National Health Insurance (NHI) categorises health care service delivery into three areas, Primary Health Care (PHC) Services, Hospital and Specialised Services, and EMS. It also recommends that standardised EMS practice should exist, determined by national norms and standards in relation to the level of care, staffing requirements, prescribed equipment, suitability of response vehicles and ambulances and other relevant components based on the level of care. The clinical teams need to have the competencies to assess, stabilise and provide essential acute emergency care and clinical interventions for categories of patients. The new EMS Regulations (December 2017) were promulgated to put in place dedicated, staffed and equipped emergency medical care (EMC), inter-healthcare facility medical treatment and transport of the ill or injured.^4^

As EMS is a component of the health system, inter-healthcare facility transport of patients is a major sub-component of the EMS, including the transfer of a critically ill neonate. Therefore, it is imperative that EMS abides by these stipulations, especially when transferring a critically ill neonate, owing to the vulnerability of this population and the life-threatening risk associated with the transfer process.

In South Africa, EMS Regulations guide the service provided by both private and public sector ambulances. The transportation of ill and/or injured patients is integral to pre-hospital and inter-facility treatment and care.^4^ The modes of transport used are ground and air ambulances, the former include response units and the latter consists of rotor wing aircraft (helicopter) and fixed wing aircraft. Although EMS in South Africa has developed rapidly over the past 20 years compared to those elsewhere in Africa and other developing countries, there is an unequal distribution of ambulance services, with many rural areas being poorly resourced. Naidoo^5^ stated that the standard of EMC offered by EMS varies widely between and within provinces, from well-developed sophisticated first-world systems to more rudimentary systems.

While the transfer of critically ill neonates has been standard practice for approximately three decades and is an integral component in the neonatal care process, it nevertheless occurs outside the neonatal intensive care units (NICU), is potentially hazardous and is associated with a very high level of risk. This places significant physiological and equipment-related stress on the neonate, who can easily deteriorate clinically during the transfer, which adversely affects its clinical outcomes.^6^ Of equal importance, although largely overlooked, is the need of the mothers of critically ill neonates.

The birth of a critically ill baby creates intense distress and anxiety for mothers who are the primary caregivers.^7,8^ This is exacerbated when their babies are transferred to other NICU for specialised treatment.^9,10^ Whyte and Jefferies^11^ wrote that the NICU environment, the transfer process and the clinical pre-hospital environment can be a frightening, confusing and difficult experience for family members. Transferring the critically ill neonate, either by ground or air, planned or emergency, results in stress and tension for both family members and health care providers.^12^ While the transfer team may cope better with the transport process, parents have a markedly different experience.^12,13^ A study by Van Manen,^13^ in Canada, revealed that transfers are associated with psychological distress and anxiety for both mothers and fathers.

Misund et al.^14^ assessed Norwegian mothers (*N* = 29) of critically ill babies over a period of 2 weeks after pre-term childbirth and found that they experienced depression due to concern about their babies’ condition. Similar findings were made in New Zealand, with mothers at the Christchurch Women’s Hospital, who reported feelings of helplessness and an inability to protect their baby from pain or painful procedures.^9^ Other studies found that mothers experienced feelings of guilt about not carrying the child to term and fears for the child’s survival.^15^

Thus far, scholarly work on how mothers experienced the neonatal transfer process in South Africa is limited. Studies, however, have found that the process of transferring a neonate for a higher level of care intensifies the depression and anxiety of family members,^12,13^ which suggests the need for greater sensitivity towards them. The study by Mullaney et al.^16^ found that of the 27 parents involved in their research, approximately 40% of parents cited separation from their baby as causing distress, 40% stated that at least one parent should accompany the baby during the transfer, 11% expressed fear that their baby would die, 26% were uncertain about what was happening to their baby, and 30% wanted reassurance that their baby ‘would be okay’. In instances where parents did not accompany their babies, 48% of parents received photographs of their babies, which created reassurance, 67% received a telephone call that the neonate had arrived safely at the receiving facility, 85% mentioned that the team had introduced themselves and 89% were given the opportunity to ask questions before the transfer. Williams et al.^17^ and Fidler and McGrath,^12^ also found that family members experienced feelings of being left out of the transfer, as they did not receive the information that they desired timeously, or sometimes not at all. Sporadic communication therefore provoked greater anxiety and confusion in the midst of acute transfers. Accurate and timeous information about the clinical condition and prognosis of the neonate should therefore be given to parents and they should be given the opportunity to ask questions.^18,19^ Rowe and Jones^20^ concluded that negative experiences during neonatal transfers can be changed by providing information regarding the neonate in a way that was easy to comprehend. They argued for sensitivity towards mothers whose cognitive functions were potentially altered by anaesthesia, fatigue and anxiety.

The National Neonatology Forum Clinical Practice Guidelines of India^21^ makes provision for providing parents with clinical and prognostic information and the anticipated time frame of the transfer and details of the receiving hospital. This includes the contact details of the NICU, along with a specific person to contact. The Transfer Policy for Neonates, Infants and Children developed by the Western Health and Social Care Trust of Ireland^22^ also recommends the same provisions. A further aspect that is overlooked relates to the clinical needs of the mother who may require medical attention during transfer.^7,23^ In South Africa the Health Professions Council of South Africa (HPCSA) clinical practice guidelines makes provision for neonatal guidelines but does not refer to the mothers. Empirical work related to the experiences of mothers during the transfer is limited, which makes this paper timeous.

The researcher was a South African educated and registered paramedic who had been employed by the EMS in the KwaZulu-Natal provincial Department of Health since 1989. His career spanned 27 years of pre-hospital care, and at the time of the study he was the Principal of the KwaZulu-Natal College of Emergency Care. During his employment, he had been involved in many inter-healthcare facility transfers of critically ill neonates using both ground and air transfers, in rural and urban areas, and had developed an interest in this area of service. More importantly, his practical experience led him to conclude that there was a disjuncture between theory and practice. He also had discovered a lack of empirical research in this field. His knowledge and expertise in this field was guided through research undertaken as part of a Master’s research study, which found that a large number of neonates are being transferred from one healthcare facility to another by non-specialised neonatal transport teams using ambulances with inadequate or malfunctioning equipment. These issues compelled him to explore the development of a programme to guide the inter-healthcare facility transfer of critically ill neonates in South Africa, to improve the overall transfer process and ensure that mortality rates were reduced. This paper, however, focuses solely on the mother within the context of the transfer. Thus, the data drawn from the neonatologists and paramedics relate solely to the experiences of the mothers as this is the crux of the study.

## Methodology

### Study design and setting

A qualitative, explorative and descriptive design was used to understand the challenges mothers faced during the emergency transfer of their critically ill neonates, and to inquire from Advanced Life Support (ALS) paramedics and neonatologists on what was better needed to prepare paramedics to deal with accompanying mothers during such emergency transfers.

### Population and sampling

The target populations for the study included the mothers from KwaZulu-Natal, and ALS paramedics and neonatologists nationally. Non-probability, sampling methods, more specifically purposive sampling, were used as a sampling strategy for all three samples.

#### Sample 1: Mothers

The mothers included in the sample were those who had used the public sector transfer EMS and were interviewed 6 months post the transfer once their babies were well. Seven mothers were interviewed. The process of data collection stopped once saturation was reached.

#### Sample 2: Advanced Life Support paramedics

Data were collected from 35 operational ALS paramedics nationally. Participants from the Free State, Gauteng, KwaZulu-Natal and Western Cape provinces who volunteered to participate were, thus, the only ALS paramedics included in the sample. Further inclusion criteria was that ALS paramedics involved in the transfers of critically ill neonates were considered. In addition, those sampled purposively were paramedics who had qualified as critical care assistants or EMC Practitioners. Furthermore, to ensure a wide range of opinions, the regions represented rural and urban areas, air and ground, and public and private ambulance services. In total, four focus group discussions were held. Each focus group consisted of eight participants; however, the focus group held in KwaZulu-Natal consisted of 11 participants.

#### Sample 3: Neonatologists

Neonatologists from across the country, who work in the public health sector were invited to participate. There were only seven neonatologists at the time of the study, found in public hospitals and, therefore, the sample and population were the same. Those included were located in hospitals in KwaZulu-Natal (KZN), Gauteng and the Western Cape.

## Data collection

Data were collected from samples 1 and 3, the mothers and neonatologists respectively, using in-depth semi-structured interviews. Semi-structured interviews enabled the researcher to collect rich data from the mothers regarding their experiences during the transfer process. Similarly, in-depth information about how the transfer process could be improved for mothers was sought from the neonatologists using in-depth interviews. Operational ALS paramedics nationally were invited to attend one of the four focus group discussions. The four regions selected for the focus group sites were the Free State, Gauteng, KwaZulu-Natal and Western Cape provinces. These regions have more established EMS and are commonly used as central points and were more accessible for the participants. Furthermore, to ensure a wide range of opinions, the regions represented included rural and urban areas, air and ground, and public and private ambulance services.

Regarding the ALS paramedics, data were drawn from focus group discussions which were considered inexpensive and stimulating and were held in order to secure data around the inter-hospital transfer (IHT) process regarding critically ill neonates. Only data regarding the challenges mothers face during the transfer process and the preparedness of the paramedics to deal with the mothers and neonatologist in the milieu of the transfer are presented in accordance with the objective of the paper.

There were two data collection instruments *viz* interview guides for samples 1 and 3 and a focus group schedule which was used to collect data from ALS paramedics. These instruments were pilot tested, with similar samples prior to use within the study, and modified based on recommendations made. In all instances the researcher collected the data and an audio tape was used to record information collected. In addition, field notes were taken.

**Interview process**: An interview schedule was used to collect data for samples 1 and 3. The questions were worded to address the issues identified through the researchers’ experience, the literature and state-of-the-art reviews, and preliminary analyses. The interview schedule was appraised by the researchers’ supervisors, who are paramedic researchers from DUT, with modifications being made as necessary. The interviews were audio recorded. The interviews lasted between 60 and 90 min.

**Focus group process:** A focus group guide was used with sample 2. A focus group moderator was selected to ensure that the researcher kept the discussion within the boundaries of the topic. The researcher had a research assistant to take notes and manage the voice recorder. The focus group discussions lasted between 90 and 120 min.

Sample 1: With regard to the recruitment process, seven mothers, who had accompanied their babies during the transfer, as established from a situational analysis of the transfer undertaken in December 2015, were contacted by the researcher and invited to participate in individual interviews. All mothers agreed to participate, and arrangements were made to meet them at the Department of Health premises for their convenience. Directions to the venue were sent to them via short message service (SMS), as most had no internet access or postal address. A room was made available at the selected facilities in which the interviews were held, with refreshments being provided on arrival. The interviews took place with a interpreter present, and confidentiality and other ethical issues were addressed at the outset.

Sample 2: Once phase 1 had been completed, the researcher forwarded a detailed letter (via email, post or fax) about the study and its requirements to operational ALS paramedics who were directly involved in neonatal transfers, inviting them to participate in a focus group discussion. This was done 3 weeks before the intended focus group discussion in their area and was accompanied by a copy of the ethics approval letter. Thereafter, the researcher made logistical arrangements in the four regions, for venues to ensure participant availability and convenience, and provided the participants with directions to the venue. The researcher also contacted the participants telephonically to advise them further about the nature of the study and what was required of them. One week before the focus group discussion, the researcher emailed and telephoned the participants to remind them of the appointment. The focus group discussions were conducted in a natural setting that was comfortable for the participants and conducive for recording. These were the boardrooms at the Department of Health premises.

Sample 3: On completion of phase 1, the researcher sent an email inviting the neonatologists identified, to participate in an interview. The participating neonatologists were generally the Heads of the NICU. Once they had accepted the invitation, the researcher travelled to meet with them at their respective institutions at their convenience. A letter of information and consent was emailed to the selected participants before the interview was conducted.

## Data analysis

The data for the three samples were undertaken using thematic analysis as per the steps outlined by Braun et al.^24^ Thematic analysis allowed the researcher to make sense of collective meanings and experiences. This helped to organise and reduce the data into themes and sub-themes. A preliminary coding scheme was generated which served as a template for the data analysis.^25^ Similar themes and recurring patterns in the data were linked together and the contrasts and differences identified.^26^

To enhance the trustworthiness of this study, the researchers made use of Guba’s model.^27^ This model provides four criteria to ascertain rigour in qualitative studies, namely credibility, dependability, conformability and transferability. Strategies used were: multiple interviews with ALS paramedics, member checking, using the triangulation process during data analysis, and maintaining an audit trail and a process journal.^28^ This ensured trustworthiness of data and reflexivity of the researcher. In addition, an expert evaluation committee was also used to validate the findings made.

## Findings made

Data derived from all three samples are presented collectively, in synergy with the objective of this paper. Five broad themes emerged from the analysis as follows:

### Theme 1: Mothers’ preparedness for the transfer

The following excerpts reflect the mother’s preparedness for the transfer:

‘I didn’t even know that my baby is being transferred. I was just sitting on this stool and watching them put all these pipes on my baby. Then these paramedics arrive in the ward with a stretcher and incubator and all the equipment.’ (Mother, interview, participant 1)

‘At the hospital I was tired and in pain, I was sleeping, then they come tell me that my baby is very sick now, but no one tell me nicely, explain what is going on. I see the ambulance people taking my baby. The nurse say go quick, quick, quick now your baby is going. I didn’t even pack my things nicely there.’ (Mother, interview, participant 7)

‘They told me mommy, now your baby is going to be transferred because it’s going to a specialist, a nice hospital. … I wait and wait. No ambulance coming.’ (Mother, interview, participant 2)

‘The ambulance people were in such a rush that they took the baby and put her in the incubator and rushing her in the ambulance, I was almost running with them while they were pushing the stretcher.’ (Mother, interview, participant 1)

Most mothers were not informed by the hospital staff that their babies were being transferred. They only became aware of the transfer when the team arrived, which resulted in shock and fear. Mothers also indicated that they had to walk or run alongside the stretcher as the neonate was being transferred to the ambulance. Empirical evidence regarding the hospitalization of a critically ill child creates considerable stress for the family.^29^ This stress is similar when a newborn requires emergency medical attention. Moreover, the crowding and busy environment associated with the NICU, where sophisticated equipment is used and diverse healthcare providers are attending to emergencies using medical terminologies unfamiliar to family members, heightens the anxiety of parents.^9,10^ Transfers commonly occur after the neonate and family are relatively settled in the NICU environment and induce greater stress when effected with little information regarding the transfer. As such, Misund et al.^14^ called for hospital staff to provide some preparedness to family members for the transfer. The fact that some mothers were compelled to walk to the ambulance, and at times even run, despite having just given birth or having undergone a caesarean section, reflects that the clinical and emotional needs of mothers are sorely overlooked.

### Theme 2: Mothers’ experiences during the transfer

#### Sub-theme 1: Lack of communication during the transfer

Most participants expressed feelings of isolation during the transfer as follows:

‘They didn’t say anything to me. They only say I must sit there on the chair inside the ambulance and they will be by hospital just now.’ (Mother, interview, participant 6)

‘There was very minimal communication, they just said that they were transporting my baby[.] Nobody was talking to me, no one was telling me what was going on, I was lost, I was left alone on the one side, as they said that they were there to treat my baby and not treat me.’ (Mother, interview, participant 3)

Moreover, they reported that their families were not informed of the transfer as evidenced below.

‘I was alone there and they not even let me phone my family. I felt so lonely and scared with all these strangers.’ (Mother, interview, participant 7)

‘My family only knew we were transferred after visiting.’ (Mother, interview, participant 5)

The use of medical jargon was also raised by participants:

‘I did not understand medical terms. Why can’t the paramedic, doctors and nurses speak simple language?’ (Mother, interview, participant 7)

The data reflected that little was communicated to both mothers and other family members regarding the transfer. This was compounded by the fact that they struggled to understand medical terms, making it difficult to understand the clinical condition of the neonate and prognosis. Other studies have documented the importance of adequate information that was communicated in a way that allayed anxiety and allowed the family to feel reassured.^16,17,18,19^ The reasons for the transfer and information regarding the receiving facility’s location, contact telephone numbers of relevant personnel, anticipated clinical procedures and estimated time of arrival is crucial information required by the family.^12^

Other studies have reported that aeromedical transfers are characterised by turbulence and altitude differences, which bring greater fear to those mothers who have not flown before and are already stressed.^16^ The pre-hospital transfer environment is characterised by adverse weather conditions, excessive noise and bumps, warning alarms of the equipment, restricted lighting and limited cabin space all of which heightens the anxiety of accompanying mothers. This increases stress, as people manage their stress by looking for relevant information to clarify and understand elements of a stressful event.

#### Sub-theme 2: Lack of confidence in the transfer team

‘There were two people working there and they looked like they didn’t know what they were doing … I feel sad.’ (Mother, interview, participant 6)

‘It seems like they don’t know what they doing because they were asking each other what do, the machine is beeping, and they were panicking … I was just crying and saying please Lord, help my baby.’ (Mother, interview, participant 7)

In addition to expressing fears regarding the competence of the paramedics, mothers were afraid that their babies would not survive. The excerpts above reflect a potential lack of preparedness and expertise of paramedics to deal with the specialised aspects of neonatal transfers.

#### Sub-theme 3: Discomfort experienced in the ambulance

‘They made me sit in the ambulance in the corner seat. There was nothing holding me, no seat belt. I did not feel safe. There was equipment all over moving around. I was very nervous and even more tense watching my baby bounce in the incubator. It was terrifying.’ (Mother, interview, participant 3)

Mothers are also unprepared for the tense and fast paced pre-hospital transfer environment. As evidenced, they experienced an uncomfortable ride in the ambulance, which prompted scholars such as Hussey et al.^30^ to suggest that they should remain seated, wear a seatbelt, be comfortable and in view of their baby. Aeromedical transfers pose greater problems for a new mother due to the noise and vibrations of aircrafts which may cause additional stress, nausea and pain. The latter may also add greater physical pain for mothers who may have had a caesarean birth or a vaginal birth which necessitated stitches.

### Theme 3: Emotional strain experienced by the mothers

The following excerpts reflected the emotional strain endured by accompanying mothers:

‘I don’t wish that experience on anyone. I felt very alone, very scared, very frightened. I didn’t even know what those machines are attached to my baby is for. I was panicking seeing the pipes all over him.’ (Mother, interview, participant 3)

‘I was feeling sad for my baby. I see my baby alone in the incubator with all these strangers. I was pregnant with my baby for nine months, I feel now they taking my baby away. I just want to hold my baby I can’t. I feel like my baby going to die now.’ (Mother, interview, participant 6)

One participant experienced being rudely removed from the ambulance because the neonate needed to be re-intubated:

‘They screamed at me and just said I must step outside the ambulance so that they can put the pipe properly … I was traumatised, I could not handle it, I just remained quiet and I prayed.’ (Mother, interview, participant 1)

Participants also experienced acute distress when they became aware that the equipment being used was dysfunctional:

‘The paramedic is not telling me the truth, the machine was not working (ventilator), and the battery was died. Now my baby is not breathing. They open the incubator putting this thing on my baby’s mouth to breathe. I was panicking, crying and please save my baby … I don’t know what was happening and was so frightened.’ (Mother, interview, participant 2)

Two other mothers endured the additional burden of being exposed to the demise of other babies in the ward, who had similar conditions to that of their own child:

‘I feel so scared and I was crying and praying and praying because at that time there was a baby died. So I was so frightened what is happening with my baby, I was saying please don’t die.’ (Mother, interview, participant 5)

Other mothers experienced feelings of guilt as they blamed themselves for their baby’s condition. They believed that their poor socio-economic circumstances had resulted in them giving birth to a sick baby. They were also saddened by the fact that they could not afford better medical care:

‘I live in a jondole. It not nice to stay here, it’s very bad. I have to do my work, cleaning, washing. I didn’t take care of my baby when I was pregnant. That why my baby is sick now. I made this baby sick. The father of my child say that I did that to my baby.’ (Mother, interview, participant 2)

‘I know it’s my fault because I am sick too, now my baby is sick too. I don’t go clinic. I got no money. That’s why my baby is sick now.’ (Mother, interview, participant 5)

All the participants reported that despite experiencing acute anxiety, the transfer team was oblivious to their distress. They said:

‘They say their job is to take care of the baby, not to look after the mother … they say me to just sit and be calm.’ (Mother, interview, participant 4)

‘They could have told me calm down, your child is fine, but they just did their own thing … I had no emotional support from the paramedics.’ (Mother, interview, participant 3)

All the mothers were found to experience acute fear, distress, anxiety, insecurity, loneliness and feelings of hopelessness during the transfer. This was exacerbated by having to endure watching the equipment and monitors being used on their babies. This was further complicated when equipment failed to work. Misund et al.^14^ concurred that news of the baby being transferred intensifies further during the transfer process itself.^9^ Hence although there is a need to prioritise the neonate, it is important that paramedics be sensitive to the trauma being experienced by mothers. This suggests educational preparedness to provide trauma counselling and the ability to attend to the clinical and comfort needs of new mothers.

### Theme 4: The clinical needs of the mother

The data reflected that the clinical needs of the mothers were often overlooked during the transfer:

‘I feel sick but nobody asked me how I’m feeling or treat me in the ambulance.’ (Mother, interview, participant 6)

‘They didn’t treat me, they didn’t even give me some pain killers. Before I went into that ambulance they take out the drip in the hospital. I was bleeding heavy and they ignore me. They didn’t do anything with me, I was feeling pain in my tummy. They ignore me, they were just busy with the baby.’ (Mother, interview, participant 7)

‘I fainted, no one treat me. I was telling them I’m sacred, I feel weak. They didn’t do anything that why I fainted … the time when I wake up, I am in the hospital. I don’t know how I get to the hospital from the ambulance. … When I wake up my baby was somewhere else. I only found out after half hour. The paramedics and the nurses was screaming me and asking why I was fainting. I said I’m fainting because I’m scared because the treatment you are doing with my child and I feel weak.’ (Mother, interview, participant 5)

While the immediate priority remains the critically ill neonate who requires constant clinical attention and continuous monitoring, it is unethical to neglect the clinical needs of the mother. It was concerning to find a total disregard for the mothers’ medical needs during the transfer. One mother reported collapsing in the ambulance and expressed that paramedics were rude towards her. Teasdale et al.^19^ affirmed that caring for a critically ill neonate during transfer is a highly skilled process that demands that the transfer team prioritise efforts related to the neonate. Mosher^7^, however, argued that within this milieu, the mother herself should be clinically stable. If not, alternative transportation should be arranged for the mother or she should remain at the hospital and be stabilised before being transferred. This calls for her to be physically assessed before the transfer to ensure that the transfer team is aware of her clinical needs and is alert to any potential clinical emergency. If it is not possible for the mother to accompany the neonate, alternate transport to the receiving facility should be provided, particularly when the condition of the neonate is critical and where the mother herself may need emergency intervention.

Latour et al.^23^ indicated the need for awareness that most transfers occur 24 h after birth while the mother is recovering from her delivery, which can prompt unforeseen clinical problems arising during a transfer. This calls for a second paramedic to be available during neonatal transfers. This can be safely accomplished only if the team consists of additional paramedics who are also able to clinically manage the mother.

### Theme 5: Preparedness of paramedics

The following excerpts were derived from data collected during interviews with neonatologists and focus group discussions with advanced life support paramedics. They called for paramedics to be better prepared to deal with mothers accompanying the transfer:

‘Counselling because you can get so caught up treating the critical baby that you forget about the mother … and social skills because it’s a stressful transfer.’ (Neonatologist, interview, participant 4)

‘We need development in that specific area, especially how to deal with the psycho-social needs of the mother … so that we can explain to them in a better and simpler way what is happening.’ (ALS Paramedic, focus group discussion 1, participant 2)

‘Often you have an anxious mom or they might even be other family members that goes along with the baby, they are scared, their baby is critical, just understanding that and empathising with them, will help them deal with the traumatic situation … so, it must be compulsory that the paramedic and the team goes for some form of training in terms of counselling.’ (Neonatologist, interview, participant 1)

‘The transfer team should constantly communicate with the escort … they won’t feel left out, they will feel as if they are part of the team. Besides it will keep their minds occupied instead of … sitting in the ambulance and having panic attacks. This way the mother will be reassured and will be comfortable with the team, instead of just jump in an ambulance with strangers, whose baby’s life [is] in their hands.’ (Neonatologist, interview, participant 6)

‘Preparation … especially the mother, because the mother normally goes with the baby, must start at the hospital. The mother must be aware of the transfer and why it is happening when it is happening, the family also must be aware of the transfer so they can support the mother. Many times when we fetch the baby, the mother is not ready, sometimes the mother is surprised that that baby is being transferred.’ (ALS Paramedic, Focus group discussion 4, participant 2)

‘On aeromedical cases we have challenges, apart from space, mothers do not want to fly because they fear the helicopter or the aeroplane, that’s why they must be well informed by the hospital staff that they are going to fly, and if they are scared of flying then this is going [to] affect them even more, or if they panic on the flight it can compromise the mission.’ (ALS Paramedic, Focus group discussion 1, participant 1)

‘I believe that the mother should be given time to call her home and tell her family about the transfer, because the family can meet her at the new hospital. I think that will make her feel more comfortable knowing that her family is meeting her there.’ (ALS Paramedic, Focus group discussion 4, participant 9).

Data from the interviews with mothers cohere with the interviews with the neonatologists and the focus group discussions with paramedics. It was evident that paramedics were inadequately prepared to help mothers deal with the trauma of the transfer. What emerged was that mothers were given little information regarding the transfer, but moreover their emotional needs were sorely overlooked as paramedics were prioritising attention towards the neonate. The acute distress mothers experienced by the impending transfer is exacerbated by anaesthetics and other drugs used during delivery and the down-surge of hormones post-delivery. These factors result in them being physiologically weak and emotionally sensitive. These findings also suggest the need for two paramedics to effect a transfer, to ensure that the mother may receive emotional support and clinical attention as evidenced within earlier excerpts which confirmed that they experience post-partum bleeding and other problems during the transfer.

## Conclusion

Holistically, these findings support the urgency for EMC education to integrate trauma counselling into a module that focuses on neonatal transfers. Current EMC education does not include education on neonatal transfers, but transfers within the context of EMC. This paper highlighted the complexities of transferring critically ill neonates within the additional context of accompanying mothers. It highlighted that there are often two patients within such a transfer, and while it is important for mothers to be part of the transfer, they must be clinically assessed to ensure that they do not present with clinical issues arising from the transfer. Potential case studies which resemble scenarios involving mothers, as in this study, should be incorporated into education. This is crucial to highlighting the clinical conditions that both mothers and neonates face. In addition to the physical conditions, it is crucial that paramedics learn to become empathic and sensitive to mothers accompanying their babies.

The transfer process is one of the most frightening, confusing and difficult experiences for mothers, which requires a more family-centred approach to neonatal transfers. Some of the most important core components that should be included as part of paramedics training is competence in basic counselling skills, and crisis stabilisation skills. The latter is crucial to help mothers remain emotionally stable during the transfer. An awareness of the context of the transfer and the fear and anxiety it potentially induces is crucial, together with the importance of collaborating with the transfer co-ordinator for support during the process. Competencies required for situations such as these transfers should include scientific knowledge about trauma, psychosocial trauma focussed assessment, trauma focussed psychosocial intervention and trauma-informed professionalism. The latter is crucial given reports from mothers that they were treated disrespectfully and with harshness during the transfer. With regard to trauma focussed psychosocial intervention, it is crucial that paramedics know how to manage mothers’ responses to the traumatic transfer process and help them remain calm and grounded despite the tensions that may arise during the transfer.

These issues point towards the need for more research around the issues challenging mothers within such transfers and also to understand what specific knowledge and skills are required to better prepare paramedics for such transfers.
